# A functional spiking model of the ITD processing pathway of the barn owl

**DOI:** 10.1186/1471-2202-12-S1-P20

**Published:** 2011-07-18

**Authors:** Victor Benichoux, Romain Brette

**Affiliations:** 1 Equipe Audition, Département d'Etudes Cognitives, Ecole Normale Supérieure, Paris, 75005, France; 2 Laboratoire Psychologie de la Perception, CNRS and Université Paris Descartes, Paris, 75006, France

## 

Sound localization in the barn owl relies on the early processing of two binaural cues, ITD (interaural time differences) and ILD (interaural level differences), that takes place in two parallel pathways in the auditory brainstem. Previous modeling studies fell into one of two classes, either they used biophysically plausible neural models on simplified binaural stimuli with artificially induced ITDs, or abstract cross-correlation models on realistic acoustical inputs. Here we present a functional spiking neural model of the ITD processing pathway of the barn owl, with a realistic virtual acoustic environment.

The acoustical environment was reproduced by filtering natural sounds through measured Head-Related Transfer Functions (HRTFs) in the barn owl (Tyto Alba). Basilar membrane filtering was modeled with a bank of linear gammachirp filters and compression. Monaural neurons in the Nucleus Magnucellularis (NM) receive inputs from auditory nerve fibers and project axons to binaural neurons in the Nucleus Laminaris (NL), with various conduction delays, so that best delays depend on preferred frequency in accordance with measured distributions [1].

Empirical findings show that the best tuning of NL neurons is invariant to level and ILD changes [3], which implies that the spike timing of their monaural inputs should also be invariant to level. This is a challenging problem, since integration to a fixed threshold inevitably results in earlier spike timing when the input level is increased. We address this issue by using a spiking model with dynamic threshold [2,4]. We derive analytical conditions under which one can achieve this level invariance, and show through numerical simulations that the responses of our model to realistic inputs are indeed robust to level and ILD changes.

The azimuth is estimated from the activation pattern of NL neurons. We found that the model could accurately estimate the azimuth of a large set of natural sounds with various levels, and significant background noise. Furthermore, we studied the robustness of ITD estimation in our model to reflective environments. We believe this study is a first step towards the development of neural models of sound localization in ecological environments.
